# Relay Architectures and Ecological Strategies of Quorum Sensing Effects on Microbial Interactions

**DOI:** 10.1111/1462-2920.70440

**Published:** 2026-10-11

**Authors:** Changchang Liang, Danlei Chen, Shuyang Sun, Aidong Yang, Jianjun Qiao, Shengbo Wu

**Affiliations:** ^1^ School of Synthetic Biology and Biomanufacturing, Key Laboratory of Systems Bioengineering (Ministry of Education), State Key Laboratory of Synthetic Biology, Tianjin University Tianjin China; ^2^ Tianjin Key Laboratory of Food Science and Health, School of Medicine Nankai University Tianjin China; ^3^ Zhejiang Institute of Tianjin University, Shaoxing Shaoxing China; ^4^ Department of Engineering Science University of Oxford Oxford UK

**Keywords:** chemical mediation, ecological interactions, environmental heterogeneity, QS‐mediated effects, quorum sensing

## Abstract

Microbial communities assemble in heterogeneous habitats, where quorum sensing (QS) influences interactions beyond signalling populations. Yet QS studies are commonly organized by signal type, circuit, or phenotype, obscuring the routes by which a QS event reaches neighbouring populations and why they prevail in particular settings. This Review synthesizes evidence from host‐associated, terrestrial, marine, and aquatic systems to develop a two‐dimensional framework for QS‐mediated ecological effects. The framework distinguishes three relay architectures by which QS activity in one population affects another: direct perception of the source signal, exposure to a QS‐regulated chemical effector, or response to a QS‐associated change in a shared host or environment. It then identifies the ecological strategies that these architectures can support, including shared signal accessibility, reciprocal coordination, asymmetric cue responsiveness, resource monopolization, and competitive or community restructuring. Across habitats, spatial structure, resource accessibility, host‐associated conditions, and temporal variability shape which relay architectures are feasible and the ecological consequences they generate. The framework positions QS as a context‐dependent process through which population‐state sensing is translated into neighbour responses, changes in habitat conditions, and community assembly. It establishes testable expectations for predicting which QS‐mediated interactions arise in a habitat and for designing interventions that steer microbiome function.

## Graphical abstract

Quorum‐sensing activity in one population affects neighbouring populations through direct signal perception, QS‐regulated chemical effectors, or host‐ or environment‐mediated relays. Across distinct habitats, these relay architectures are associated with ecological strategies ranging from shared signal accessibility and reciprocal responsiveness to resource monopolization, niche restructuring, and associational resistance.

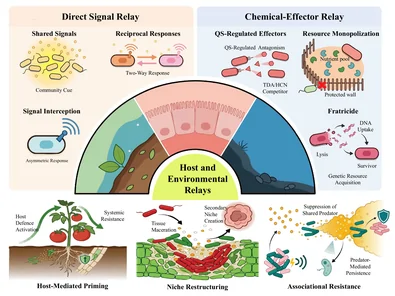

## 1 Introduction

Microorganisms rarely exist as independent entities in natural environments. Instead, they assemble into highly diverse and dynamic communities including soil, oceans, sediments, wetlands, hydrothermal vent, plant rhizosphere, and numerous other environmental niches (Orsi 2018; Qian et al. 2022; Raina et al. 2022; Philippot et al. 2024). Within these communities, cooperation, competition, nutrient exchange, chemical antagonism, and habitat modification jointly determine community assembly, ecosystem functioning, and biogeochemical cycling across spatial and temporal scales (De Wit et al. 2022). Quorum sensing (QS) provides an important regulatory entry point into these processes because it links extracellular chemical cues to population‐level changes in gene expression (Papenfort and Bassler 2016; Liu et al. 2025). However, the ecological influence of QS extends beyond cells that produce and detect the same signal. A QS event in one population may be perceived directly by another population, converted into a QS‐controlled metabolite, relayed through a modified host or environmental condition.

QS is a cell‐density‐dependent regulatory system in which microorganisms synthesize, release, and perceive diffusible signalling molecules, allowing coordinated regulation of gene expression once signalling molecules accumulate above threshold concentration. QS systems are chemically diverse and have been identified across a wide range of microorganisms, with major signalling families including N‐acyl homoserine lactones (AHLs) and diffusible signal factors (DSFs) in many Gram‐negative bacteria, autoinducing peptides (AIPs) in Gram‐positive bacteria, and universal signals such as autoinducer‐2 (AI‐2), which can be detected by phylogenetically distinct bacterial populations (Papenfort and Bassler 2016). Through this mechanism, QS controls a broad spectrum of phenotypes closely associated with microbial ecology, including biofilm formation, production of extracellular enzymes and secondary metabolites, motility, nutrient acquisition, stress adaptation, and interactions with neighbouring organisms (Liu et al. 2025; Zeng et al. 2023). These QS‐regulated phenotypes frequently determine how a microbial population affects its neighbours. By altering competitive interactions, resource availability, chemical exposure, or local habitat conditions, they can reshape the ecological context experienced by surrounding microorganisms and, at the community scale, influence microbial succession, rhizosphere colonization, marine biogeochemical cycling, and the assembly of complex microbiomes (Qian et al. 2022; Berendsen et al. 2012; Zhao et al. 2025).

Consequently, extensive efforts over the past several decades have elucidated the molecular diversity of QS systems, their regulatory networks, and the physiological functions they control across a wide range of environmental microorganisms (Avcı et al. 2026; Letizia et al. 2025; Wu et al. 2023). Despite these advances, our understanding of the ecological significance of QS remains fragmented. Existing studies have predominantly focused on individual signalling systems, specific microbial taxa, or particular ecological phenomena, such as interspecies signal exchange, QS‐regulated secondary metabolites, host‐associated interactions, and biofilm development. Although these studies have substantially expanded our mechanistic understanding of QS, they are generally interpreted independently according to their molecular mechanisms or ecological contexts, making it difficult to integrate diverse observations into a coherent ecological perspective (Letizia et al. 2025; Paharik et al. 2017). Increasing evidence indicates that the ecological consequences of QS are not determined solely by signal perception, but also by the routes through which QS‐regulated activity in one population affects another. Depending on ecological context, these effects can involve direct perception of the source signal, exposure to a QS‐regulated effector, or response to a host or environmental condition modified by QS activity. Although these mechanisms have been extensively investigated, they are generally studied as independent phenomena, making it difficult to establish an integrated ecological perspective on how QS shapes microbial communities across natural environments. The challenge is particularly apparent across heterogeneous habitats, where spatial organization, resource availability, and host association shape the settings in which these routes operate. A framework that places these pathways within a common ecological logic is therefore needed.

In this review, we organize these interactions along two complementary dimensions. The first identifies three relay architectures. Here, relay refers to the extension of a QS‐regulated effect beyond its source population, whereas architecture denotes the biological, chemical, or environmental link through which that effect reaches another population. Accordingly, a receiver may respond directly to the source signal, to a QS‐regulated chemical effector, or to a shared host or environmental condition modified by QS activity. These alternatives define three relay architectures: direct signal perception, chemical‐effector mediation, and host‐ or environment‐mediated propagation. The second identifies the ecological strategies associated with these relay architectures, including shared signal accessibility, reciprocal coordination, asymmetric cue responsiveness, resource monopolization, and competitive or community restructuring. Across terrestrial, aquatic, marine, and host‐associated systems, environmental heterogeneity shapes the contexts in which particular relay architectures and strategies can operate. Together, these complementary dimensions provide a unified conceptual perspective for interpreting the ecological complexity of quorum sensing across environmental microbial communities.

## 2 Direct Perception of QS Signals

Direct signal perception represents the relay architecture in which a signal released by one microbial population is perceived by a receptor in another population and changes the receiver's regulatory state. The initiating signal itself therefore provides the connection between source and receiver, without requiring a QS‐regulated antibiotic, public good, host response, or environmental alteration as an intermediate. This mode encompasses broadly accessible shared signals, reciprocal crosstalk between signal‐producing partners, and asymmetric signal interception by organisms that respond to signals produced by others. These strategies differ in directionality and ecological outcome, but all retain the same basic organization in which a source‐derived signal is directly perceived by a neighbouring microbial population.

### 2.1 Shared Signals Provide Ecologically Accessible Cues

The most direct relay architecture occurs when a QS signal produced by one microbial population can be detected by multiple neighbouring populations. Whereas some QS systems are highly specific, shared signals can be detected by organisms with compatible receptors or uptake systems. Detection by several populations does not imply uniform responses, because signal availability and responsiveness depend on local cell density, spatial confinement, signal stability, and the receptor repertoire of potential populations. Shared signals can therefore act as chemical cues associated with local population activity or composition.

AI‐2 illustrates this principle particularly well. Produced through the LuxS pathway in many bacterial taxa, AI‐2 can be released into extracellular environments and detected by microorganisms with distinct taxonomic backgrounds. For example, *Vibrio harveyi* synthesizes AI‐2 as part of its QS system. Because AI‐2 is not specific to a single producer species, its extracellular availability can reflect local microbial activity, to which compatible populations may show distinct regulatory responses (Liu et al. 2025; Su and Ding 2023).

The ecological significance of shared signals becomes particularly evident when AI‐2 produced by one population elicits a response in a compatible neighbouring population without being specific to that producer species. In saliva‐fed dual‐species biofilms, AI‐2 produced by *Streptococcus oralis* was required for high‐biomass mutualistic growth with *Actinomyces naeslundii* , and this response occurred only within a restricted AI‐2 concentration range (Rickard et al. 2006). Likewise, AI‐2 released by *Enterococcus faecalis* enabled *Escherichia coli* to maintain Lsr‐dependent AI‐2 responsiveness, chemotax towards *E. faecalis* aggregates, and initiate mixed‐biofilm formation at cell densities insufficient for this behavior in monoculture (Laganenka and Sourjik 2018). Together, these observations indicate that extracellular AI‐2 concentrations are associated with the activity and spatial organization of neighbouring microbial populations. They reflect local signal production, uptake and degradation and can couple aggregation and biofilm formation to local community conditions.

A host‐associated example demonstrates that a shared AI‐2 pool can directly alter community assembly. During recovery from cholera, *Blautia obeum* expands in the gut community and increases *luxS* expression and AI‐2 production following *Vibrio cholerae* invasion. In gnotobiotic mice, *B. obeum* restricts *V. cholerae* colonization, and its AI‐2 represses several *V. cholerae* colonization and virulence factors through a pathway independent of the canonical AI‐2 receptor LuxP. Rather than broadly inhibiting growth through altered iron acquisition or biofilm formation, this case shows that *B. obeum*‐derived AI‐2 can repress *V. cholerae* colonization‐ and virulence‐associated factors and reduce colonization capacity during recovery from cholera (Hsiao et al. 2014).

Collectively, these examples show that a QS molecule can become an ecologically accessible cue when it is produced by one population and detected by more than one taxon. Such shared accessibility can link local signal availability to regulatory responses in neighbouring populations without reciprocal signalling or a common regulatory outcome. Its ecological significance lies in expanding the range of populations that can respond directly to a source‐derived signal within a shared microsite.

### 2.2 Reciprocal Signal Responsiveness Coordinates Interpopulation Responses

Reciprocal signalling occurs when neighbouring microbial populations both produce and perceive signals to which the other population responds. In contrast to shared signal accessibility, in which a signal produced by one population can be detected by several compatible populations, reciprocal signalling involves two populations that each contribute to and respond to the local signal pool. This bidirectional responsiveness can couple regulatory states across populations without requiring identical signals, responses, or ecological outcomes.

A representative example of reciprocal signalling is provided by interactions between *Pseudomonas aeruginosa* and *Burkholderia multivorans* , two opportunistic pathogens that can co‐occur during polymicrobial infection. Both species possess LuxI‐type synthase and LuxR‐type receptor QS circuits, and culture‐based reporter assays have shown that the RhlR receptor of *P. aeruginosa* and the CepR receptor of *B. multivorans* can each recognize an AHL produced by the other species. Genetic and transwell coinfection experiments further demonstrated that this interspecies crosstalk contributes to the enhanced virulence of mixed infections in a *Caenorhabditis elegans* model (Slinger et al. 2024). This system therefore provides direct evidence that compatible QS networks can connect otherwise independent microbial populations: a signal released by one species can be perceived by the other and alter its regulatory state, while both partners retain the capacity to modify the shared signalling environment through their own QS activities. Although the temporal dynamics of this interaction have not yet been resolved at single‐cell or infection‐site scales, the reciprocal cross‐recognition of AHL signals establishes the mechanistic basis for bidirectional signal responsiveness during coinfection (Slinger et al. 2024).

Reciprocal responsiveness to heterologous AHLs can also influence biofilm‐associated interactions between *Acinetobacter baumannii* and *Pseudomonas aeruginosa* , two opportunistic pathogens that may occupy the same infection sites. Both species possess AHL‐based quorum‐sensing systems, and in vitro experiments with AHL‐synthesis‐defective mutants showed that the biofilm phenotype of each species could be restored or enhanced by the addition of AHLs produced by the other species. These findings demonstrate that AHLs released by one population can elicit a biofilm‐associated response in a neighbouring population and can modify its biofilm‐associated behaviour, even when the receiver does not produce the relevant signal itself (Bhargava et al. 2012). Thus, rather than functioning as completely insulated signalling systems, the two QS networks exhibit heterologous responsiveness that can couple biofilm development across species boundaries. Although the spatial and temporal dynamics of this interaction remain to be resolved in natural polymicrobial communities, this system provides experimental evidence that compatible AHL responses can enhance dual‐species biofilm formation in vitro.

Together, these examples show that reciprocal signalling is distinguished from shared signal accessibility by bidirectional signal production and perception. When both populations contribute to and respond to the local signal pool, their regulatory states can become coupled, linking QS‐controlled phenotypes such as biofilm formation or virulence‐associated traits across coexisting populations. The ecological strategy is therefore reciprocal coordination, in which local co‐occurrence and compatible QS systems permit coordinated interpopulation responses without requiring identical signals, regulatory outputs, or ecological consequences.

### 2.3 Signal Interception Shapes Responses to Neighbouring Populations

Signal interception occurs when a population detects a QS signal produced by a neighbouring population without producing the same or a functionally equivalent signal itself. This differs from reciprocal signalling because the responding population does not contribute to the corresponding local signal pool. In this context, the heterologous QS signal acts as a chemical cue whose detection can alter regulatory or physiological responses when compatible signal producers and receivers co‐occur.

A well‐defined example of asymmetric AHL sensing occurs in the bovine gastrointestinal tract, where enterohemorrhagic *Escherichia coli* (EHEC) detects AHLs produced by other microorganisms through the orphan LuxR‐family receptor SdiA. EHEC does not rely on an endogenous AHL synthase for this response; instead, SdiA detects exogenous AHLs that are abundant in the bovine rumen but were not detected in other regions of the gastrointestinal tract. In response to rumen‐derived AHLs, SdiA represses expression of the locus of enterocyte effacement and Type III secretion‐associated genes while activating the glutamate decarboxylase (Gad) acid‐resistance system. This regulatory switch promotes adaptation to a commensal lifestyle in the rumen, and competition experiments in cattle demonstrated that SdiA contributes to EHEC colonization of this defined host‐associated niche (Hughes et al. 2010). Rather than broadly monitoring all surrounding Gram‐negative bacteria, EHEC therefore uses SdiA to identify a specific chemical environment associated with its natural bovine reservoir and to adjust its physiological program accordingly. The rumen‐restricted distribution of these AHLs links SdiA‐dependent regulation to a defined host‐associated chemical environment.

In multispecies plant lesions, *Pectobacterium wasabiae* can respond to AHLs produced by neighbouring soft‐rot bacteria before its own population reaches the AHL threshold. Its broad‐specificity receptor ExpR2 responds to cognate and heterologous AHLs, increases expression of the regulatory RNA *rsmB*, and thereby bypasses the usual Gac/Rsm requirement for activating plant‐cell‐wall‐degrading functions. In vivo, this cross‐species response enabled earlier virulence induction in lesions containing AHL‐producing bacteria (Valente et al. 2017). Ecologically, heterologous AHLs can act as chemical cues that tissue degradation has already been initiated by neighbouring bacteria. The resulting strategy is threshold adjustment: *P. wasabiae* can activate tissue‐degradation functions in an already developing lesion rather than relying solely on its own cell‐density signal.

On leaf surfaces, resident epiphytic bacteria can alter the behaviour of the plant pathogen *Pseudomonas syringae* through heterologous AHL production. Approximately 7% of leaf epiphytes isolated in this system produced the cognate *P. syringae* signal *N*‐(3‐oxohexanoyl)‐l‐homoserine lactone (3OC6‐HSL), often at concentrations exceeding those produced by the pathogen itself (Dulla and Lindow 2009). Premature activation of the pathogen's QS response suppressed swarming motility and reduced lesion formation on leaves, whereas AHL degradation abolished this protective effect. This example illustrates a potential cost of heterologous cue responsiveness rather than directed antagonism by epiphytes: a chemical cue that activates QS at an inappropriate time can shift *P. syringae* to a phenotype poorly suited to water‐limited leaf surfaces.

Signal interception can also link microbial responses to competitive conditions within complex host‐associated communities. During intestinal colonization, *Vibrio cholerae* produces the cholera autoinducer‐1 (CAI‐1) signal through the CqsA synthase‐CqsS sensor system, which reflects population density and regulates transitions between different ecological states. Enteropathogenic *Escherichia coli* (EPEC) responds to *V. cholerae* ‐derived CAI‐1, which may serve as an early chemical cue of local *V. cholerae* density and associated community conditions. By responding to a cue produced by another species rather than relying exclusively on its own population‐density signals, EPEC may modulate virulence‐associated responses during co‐occurrence (Gorelik et al. 2019). This response therefore represents a potential strategy of context‐dependent virulence modulation.

Signal interception may be particularly consequential in spatially structured habitats where the same microbial populations repeatedly co‐occur, because repeated co‐occurrence can make heterologous signals reliable chemical cues of local partner presence. *Stenotrophomonas maltophilia* and *Pseudomonas aeruginosa* are environmental generalists that can occupy shared niches, including plant rhizospheres and host‐associated biofilms such as cystic fibrosis airways. In mixed‐species biofilms, DSF‐family signals produced by *S. maltophilia* are detected by the *P. aeruginosa* sensor kinase PA1396, despite the fact that *P. aeruginosa* does not produce the same signal. DSF perception alters the architecture of *P. aeruginosa* biofilms and increases the abundance of stress‐tolerance‐associated proteins, resulting in enhanced tolerance to polymyxin (Ryan et al. 2008).

The ecological relevance of this interaction lies in the fact that DSF produced by a recurrent neighbouring population can provide an early chemical cue of *S. maltophilia* presence. In response to DSF, *P. aeruginosa* reorganizes its biofilm and increases stress tolerance, including tolerance to polymyxin. In dense, surface‐associated communities, stable spatial proximity may make this cue sufficiently reliable to support stress preconditioning before direct antimicrobial exposure. Although direct in situ demonstration in cystic fibrosis sputum remains unavailable, this system provides a mechanistically resolved model for how DSF‐mediated signal interception can connect community composition with stress adaptation in polymicrobial habitats.

Responses to structurally diverse QS signals can also vary among microbial populations. In *Streptococcus pneumoniae* , polymorphic BlpC peptide pheromones and diversified BlpH receptors generate differential sensitivity patterns among strains. Some populations preferentially respond to non‐cognate pheromones produced by neighbouring lineages, allowing bacteriocin production to be initiated before activation by their own signalling systems. This selective responsiveness shows that asymmetric signal responsiveness can depend on both signal structure and receptor specificity (Miller et al. 2018).

Together, these examples show that signal interception has a distinct ecological role within direct QS‐signal perception. Unlike shared signal accessibility or reciprocal signalling, it produces a one‐sided response: in spatially structured communities where populations repeatedly co‐occur, a heterologous QS signal can serve as a chemical cue of local population presence or habitat condition. This asymmetric cue responsiveness can shift competitive phenotypes without requiring corresponding signal synthesis, although its ecological value depends on cue reliability and the cost of the induced response.

## 3 Mediation by QS‐Regulated Effectors

Mediation by QS‐regulated effectors represents a relay architecture in which QS‐regulated activity in a source population generates extracellular effectors that influence neighbouring populations. Neighbouring populations need not perceive the initiating QS signal itself; instead, they encounter QS‐associated antibiotics, redox‐active metabolites, extracellular enzymes and their released products, bacteriocins, volatile compounds, or contact‐dependent effectors. The ecological outcome depends on the transport and persistence of the mediator, the physiological target in the receiver, and local resource conditions. This mode of propagation can thereby link population sensing to suppression, metabolic reprogramming, resource release, and survival selection.

### 3.1 QS‐Regulated Effectors Shape Interpopulation Competition

Beyond direct perception of QS signals, microbial populations can propagate QS activity through chemically active metabolites. In this mode of propagation, QS does not directly act as the ecological effector; instead, it functions as an upstream regulatory system that coordinates the production of extracellular compounds whose activities reshape neighbouring microbial populations. Thus, QS‐regulated extracellular effectors connect population‐level QS activity to chemically mediated effects on neighbouring populations.

A well‐resolved example of quorum‐sensing‐regulated chemical translation is provided by *Phaeobacter inhibens* DSM 17395. In this organism, the AHL synthase PgaI produces *N*‐(3‐hydroxydecanoyl)‐l‐homoserine lactone (3OHC10‐HSL), and AHL‐dependent regulation activates *tdaA* and tropodithietic acid (TDA) biosynthesis (Berger et al. 2011). This regulatory architecture links investment in an extracellular antibiotic to a local density threshold rather than to the mere presence of potential competitors. The ecological relevance of this density‐dependent regulation is most plausible in surface‐associated marine microhabitats, including host‐ and particle‐associated patches, where restricted mixing can retain both cells and diffusible metabolites (Berger et al. 2011). At inhibitory concentrations, TDA suppresses susceptible neighboring populations. At lower concentrations, TDA also alters global gene expression in the producer, including genes associated with motility, attachment, and biofilm formation (Beyersmann et al. 2017). This case illustrates how QS‐regulated antibiotic production can couple population density to local community structuring in surface‐associated marine habitats (Berger et al. 2011; Beyersmann et al. 2017; Urvoy et al. 2022).

The interaction between *Burkholderia thailandensis* and *Chromobacterium subtsugae* CV017 provides a tractable model of QS‐regulated antagonism coupled to QS‐regulated defence. Both organisms are soil‐associated saprophytes, making heterogeneous, locally crowded soil microhabitats a plausible ecological setting; however, the available evidence derives from laboratory co‐culture rather than directly in natural soil communities (Loo et al. 2023). In *B. thailandensis* , the BtaR2–BtaI2 AHL circuit activates bactobolin biosynthesis, thereby delaying investment in an energetically costly antibiotic until a local population threshold has been reached (Duerkop et al. 2009). Exposure to bactobolin does not simply eliminate the competing *Chromobacterium* population: in *C. subtsugae* , its own QS system increases expression of the CdeAB–OprM efflux pump and thereby raises bactobolin resistance (Evans et al. 2018). The resulting interaction is therefore not a one‐way chemical attack, but a density‐dependent competitive feedback in which the producer coordinates antagonism while the receiver coordinates defence.

*Pseudomonas aeruginosa* and *Staphylococcus aureus* are frequently co‐isolated from chronic infections, including cystic‐fibrosis‐associated airways (Hoffman et al. 2006). In *P. aeruginosa* , 4‐hydroxy‐2‐heptylquinoline N‐oxide (HQNO) production is embedded within the Pseudomonas quinolone signal (PQS) alkylquinolone QS network. 2‐Heptyl‐4‐hydroxyquinoline (HHQ) and PQS activate the transcriptional regulator PqsR (MvfR), which promotes *pqsABCDE* expression through a positive‐feedback loop; PqsL, together with the *pqsABCD* gene products, is required for the biosynthesis of HQNO. HQNO should therefore be considered a QS‐associated downstream alkylquinolone output rather than the cognate transcriptional signal of PqsR (Rampioni et al. 2016). At physiologically relevant concentrations, HQNO inhibits respiratory metabolism in *S. aureus* , and prolonged exposure can select slow‐growing, respiration‐defective small‐colony variants. These variants display increased aminoglycoside tolerance and enhanced persistence (Hoffman et al. 2006; Fugère et al. 2014). This interaction is therefore better interpreted as metabolic selection rather than simple competitor elimination: QS‐associated HQNO production can shift *S. aureus* towards a persistent physiological state that permits long‐term coexistence in the spatially structured and treatment‐exposed airway environment.

QS‐dependent chemical antagonism is not restricted to host‐associated communities but is widespread in environmental microbial habitats. In the rhizosphere, where microorganisms compete intensely for limited resources and spatial opportunities, QS‐regulated regulatory networks in *Pseudomonas* species coordinate the production of diverse antimicrobial metabolites, including phenazines, hydrogen cyanide (HCN), and pyrrolnitrin. Rather than representing independent inhibitory events, coordinated regulation of multiple biosynthetic pathways enables bacterial populations to control when and where costly chemical defenses are deployed. This strategy illustrates how activity can be amplified through downstream metabolic networks, converting local population‐level signals into broader chemical effects that influence microbial community dynamics in soil environments (Msimbira et al. 2026).

Collectively, QS‐regulated chemical effectors represent a relay architecture in which an extracellular metabolite, rather than the source signal itself, acts on another population. These effectors can suppress neighbouring populations, shift their physiology, or restrict access to locally released resources, thereby extending QS effects beyond direct signal perception.

### 3.2 Resource Monopolization Redirects Chemical QS‐Mediated Effects

Resource monopolization occurs when QS regulation couples the release of nutrients from a shared substrate to the restriction of access to those nutrients. In this strategy, the source population does not merely produce an extracellular activity that benefits nearby cells. Instead, QS coordinates substrate depolymerization with traits that preserve the resulting resource for the producer population. This mechanism has been resolved in a defined casein coculture model involving *Pseudomonas aeruginosa* and *Burkholderia multivorans* . The model provides mechanistic evidence for a QS‐regulated interaction, but it should not be interpreted as a direct reconstruction of either a soil or airway community (Smalley et al. 2015).

In casein medium, LasI‐derived 3‐oxo‐C12‐HSL activates LasR‐dependent production of extracellular elastase in *P. aeruginosa* . Elastase hydrolyses casein and releases diffusible peptides and amino acids, thereby converting an initially inaccessible polymeric substrate into a shared nutrient pool. Under these conditions, *B. multivorans* does not efficiently hydrolyse casein itself, but can grow when elastase is supplied and can use nutrients released through *P. aeruginosa* proteolysis.

QS regulation simultaneously limits unrestricted exploitation of this newly accessible nutrient pool. Hydrogen cyanide, rhamnolipids, and phenazines each contribute to the competitive performance of *P. aeruginosa* in the casein coculture model, with hydrogen cyanide making the largest individual contribution. Hydrogen cyanide production in *P. aeruginosa* is itself under QS control, directly linking population‐density sensing to the chemical restriction of access to the nutrients released through extracellular proteolysis (Smalley et al. 2015). During serial passage, wild‐type *P. aeruginosa* nearly eliminates *B. multivorans* , whereas an antimicrobial‐deficient mutant continues to release proteolysed nutrients but permits *B. multivorans* to persist at appreciable frequency. Resource monopolization therefore redirects QS‐derived chemical propagation into an asymmetric nutrient landscape. QS links the costly opening of a polymeric resource to chemical exclusion, allowing the producer to retain a disproportionate share of the benefit that it has created.

### 3.3 QS‐Regulated Lysis Links Competition to Genetic Resource Acquisition

In *Streptococcus pneumoniae* , competence‐associated fratricide provides one route by which QS‐regulated lysis couples competition to genetic acquisition. When the competence‐stimulating peptide reaches the response threshold, ComD–ComE signalling activates the competence programme and the alternative sigma factor ComX. Competent cells express the cell‐wall hydrolase CbpD and the immunity protein ComM, whereas neighbouring non‐competent cells remain vulnerable to lysis (Steinmoen et al. 2003; Kausmally et al. 2005). In pneumococcal biofilms, CbpD‐dependent fratricide increases the availability of donor DNA and substantially enhances gene transfer between neighbouring pneumococci. This strategy is ecologically relevant to nasopharyngeal and oropharyngeal biofilms, where *S. pneumoniae* coexists with closely related commensals, including *Streptococcus mitis* and *Streptococcus oralis* , and where homologous DNA is more likely to be retained after uptake (Wei and Håvarstein 2012). A mechanistically distinct example occurs on chitinous surfaces in aquatic habitats colonized by *Vibrio cholerae* . Chitin sensing activates TfoX, whereas high cell density is integrated through the CAI‐1/AI‐2 quorum‐sensing pathway and the regulator HapR. Together, these inputs promote QstR‐dependent expression of both DNA‐uptake functions and the Type VI secretion system (T6SS) (Borgeaud et al. 2015; Jaskólska et al. 2018). The T6SS kills neighbouring non‐immune cells, releasing DNA that can subsequently be taken up by competent *V. cholerae* cells (Borgeaud et al. 2015). Transferred DNA can include functional T6SS effector and immunity genes, enabling recipient populations to alter their subsequent competitive capacity (Thomas et al. 2017). QS therefore acts here as a high‐density regulatory gate that couples ecological opportunity on chitin surfaces with neighbour killing and genetic resource acquisition.

## 4 Relays Through QS‐Modified Host and Environmental Conditions

Host‐ and environment‐mediated relays represent relay architectures in which QS‐regulated activities modify a shared host or environmental context before another population experiences the resulting conditions. In this route, neighbouring populations respond not to the initiating signal or a single chemical effector, but to altered conditions such as host responses, nutrient availability, biofilm architecture, spatial structure, or predator activity. These changes can reshape colonization opportunities, community composition, and the relative performance of neighbouring microorganisms.

### 4.1 Host‐Mediated Propagation of QS‐Derived Ecological Effects

QS activity can influence microbial populations indirectly when microbial populations are affected not by QS signals or QS‐regulated metabolites themselves, but by changes occurring within a shared host environment. In this propagation route, hosts function as biological intermediaries through which QS‐regulated microbial activities induce physiological responses, which subsequently reshape the ecological conditions experienced by surrounding microbial populations. Thus, QS activity can influence host‐associated ecosystems through host‐mediated processes rather than direct signal perception.

A defined rhizosphere‐to‐phyllosphere example is provided by *Serratia liquefaciens* MG1 and tomato plants. *S. liquefaciens* MG1 colonizes tomato roots and produces AHLs in the rhizosphere, whereas the isogenic AHL‐deficient mutant MG44 is impaired in its ability to protect plants from the leaf pathogen *Alternaria alternata* (Schuhegger et al. 2006). Root colonization by the AHL‐producing strain increases salicylic acid accumulation in leaves and induces salicylic acid‐ and ethylene‐responsive defence genes, including pathogenesis‐related protein 1a and chitinase genes; these responses are not detected after inoculation with the AHL‐deficient mutant (Schuhegger et al. 2006). Consequently, reduced fungal growth and disease symptoms occur in leaves that are spatially separated from the root‐associated bacterial population (Schuhegger et al. 2006). This system should therefore be interpreted as AHL‐dependent host priming, rather than as a general consequence of all QS‐regulated traits of *S. liquefaciens* . Ecologically, this host‐mediated strategy couples the establishment of an AHL‐producing root‐associated population to a host‐wide defensive state, thereby constraining a spatially separated foliar pathogen without direct bacterium–fungus contact.

A related host‐mediated route is suggested by the rhizobial symbiont *Sinorhizobium meliloti* . This soil‐associated bacterium produces long‐chain *N*‐(3‐oxotetradecanoyl)‐l‐homoserine lactone (oxo‐C14‐HSL) during plant‐associated growth. In an *Arabidopsis* model, inoculation with oxo‐C14‐HSL‐producing *S. meliloti* increased resistance to the foliar bacterial pathogen *Pseudomonas syringae* , whereas an otherwise comparable strain expressing an AHL lactonase did not confer the same protection (Zarkani et al. 2013). This in planta system supports the interpretation that AHL production in a root‐associated bacterial population can be integrated into a host defensive state that subsequently constrains a spatially separated microbial population. The ecological implication is that root‐associated rhizobia may influence pathogen establishment or disease development beyond the local diffusion range of their own signals.

A second example is provided by *Serratia plymuthica* HRO‐C48, a bacterium originally isolated from the oilseed‐rape rhizosphere. Its AHL system contributes to root colonization and to plant‐protective traits. Disruption of AHL signalling reduced systemic resistance to *Botrytis cinerea* in bean and tomato and weakened protection against damping‐off caused by *Pythium aphanidermatum* (Pang et al. 2009). Because the systemic‐resistance assays separate the root‐associated bacterial population from the challenged fungal pathogen, this aspect of the interaction is best understood as host‐mediated limitation of pathogen performance rather than direct bacterial antagonism. Together with the *Serratia liquefaciens* –tomato–*Alternaria* system, these examples indicate that plant‐associated QS activity can influence microbial establishment across compartments through host immune and physiological relays.

Host‐mediated propagation highlights that hosts are not merely passive habitats for microbial communities but active ecological components that integrate microbial signals with immune, metabolic, and physiological processes. Through this intermediary role, QS‐regulated microbial activities can influence populations that never directly encounter the original QS signals, expanding the ecological reach of QS activity beyond direct microbial interactions.

### 4.2 Host‐Associated Niche Restructuring Alters Microbial Opportunities

Beyond host‐mediated effects, QS activity can also propagate through modification of the surrounding physical and chemical environment. In this route, QS‐regulated microbial activities reshape ecological conditions, including spatial organization, extracellular matrix structure, and local resource accessibility. Neighbouring populations are therefore influenced not through direct perception of QS signals or metabolites, but through the altered habitat conditions generated by QS‐controlled collective behaviours.

Host‐associated niche restructuring occurs when QS‐regulated activity in a source population modifies host tissue or host‐derived resources, and the resulting host condition changes the opportunities available to another microbial population. In this relay architecture, the receiving population is affected by the reconstructed host‐associated niche rather than by the original QS signal. A well‐resolved natural example occurs in plant soft‐rot pathosystems. In soft‐rot pectobacteria, AHL‐dependent QS controls the coordinated production and secretion of plant cell wall‐degrading enzymes together with additional virulence functions that promote host tissue maceration and disease development (Liu et al. 2008). As infection progresses, this QS‐regulated activity converts intact plant tissue into a chemically and physically altered lesion environment enriched in released nutrients and characterized by modified local conditions. In this context, the relevant ecological relay is not direct signal perception by a neighbouring population, but the host‐derived niche that emerges after QS‐dependent tissue breakdown. The downstream consequence of this niche construction has been demonstrated for enteric bacteria in tomato and leafy‐tissue soft rot. *Salmonella enterica* serovar Typhimurium reaches much higher population sizes in tomatoes undergoing *Pectobacterium*‐mediated soft rot than in intact tissues, and this benefit depends on plant maceration rather than on the mere presence of the soft‐rot pathogen (George et al. 2018). Transcriptomic and genetic analyses further indicate that *Salmonella* exploits the nutrient‐rich and physiochemically altered conditions generated within the lesion. A similar enhancement has also been reported for *Escherichia coli* O157:H7 on soft‐rotted leaves. Taken together, these studies support a host‐mediated propagation route in which QS in the source pathogen promotes tissue collapse, the resulting lesion becomes a secondary ecological niche, and neighbouring bacterial populations subsequently benefit from that reconstructed environment rather than from the original signal itself (Liu et al. 2008; George et al. 2018; Kwan et al. 2013).

A second plant‐associated example is provided by *Dickeya dadantii* and *Escherichia coli* O157:H7 on postharvest lettuce. In *D. dadantii* , the Vfm quorum‐sensing system regulates the production of pectate lyases, which are secreted to degrade plant cell walls (Banerjee et al. 2022). In lettuce coinoculation assays, *D. dadantii* promoted proliferation of *E. coli* O157:H7, whereas a mutant defective in Type II secretion of pectate lyases lost this effect; higher pectate‐lyase activity was correspondingly associated with greater *E. coli* proliferation (Yamazaki et al. 2011). Together, these findings support a host‐associated relay in which QS‐regulated pectinolysis converts leaf tissue into a macerated niche that favours the growth of a neighbouring bacterial population. The relevant connection is therefore mediated by the altered plant tissue, rather than by direct perception of the initiating QS signal by *E. coli* . This case illustrates how QS‐dependent host tissue breakdown can create secondary colonization opportunities in plant‐associated habitats.

Together, these plant‐associated systems indicate that host mediation can expand the ecological reach of QS‐associated activity beyond the local diffusion range of a bacterial signal or effector. The relevant strategy is not simply host colonization, but QS‐regulated modification of host tissue or host‐derived resources that changes the conditions under which other microbial populations establish and proliferate.

### 4.3 Predator‐Mediated Effects Alter the Persistence of Neighbouring Populations

QS‐derived ecological effects can propagate through biotic components of the local environment, including bacterivorous predators. Protozoan grazing is a major source of mortality for bacteria in natural biofilms and can therefore determine which bacterial populations persist within surface‐associated communities. In a three‐member biofilm containing *Pseudomonas aeruginosa* , *Klebsiella pneumoniae* , and *Pseudomonas protegens*, grazing by *Tetrahymena pyriformis* strongly reduced monospecies biofilms of *K. pneumoniae* and *P. protegens*, whereas their survival increased markedly when they co‐occurred with *P. aeruginosa* (Goh et al. 2023).

Mechanistic analysis showed that rhamnolipids produced by *P. aeruginosa* were the major anti‐grazing factor, while the PQS pathway provided an additional protective component: simultaneous disruption of rhamnolipid production and PQS biosynthesis abolished protection, and exogenous PQS restored toxicity towards the predator. Thus, the ecological effect of the *P. aeruginosa* QS‐associated programme was not restricted to the producer population. By suppressing a shared predator, it indirectly created a protective biofilm microenvironment in which otherwise grazing‐sensitive neighbouring populations could persist. This represents an ecological strategy of associational resistance, in which QS‐derived outputs reduce shared predator pressure and thereby stabilize multispecies coexistence rather than simply inhibit a competitor.

The consequences of such QS‐mediated modification of environmental conditions can subsequently extend to the evolution of signalling‐associated traits. In a polymicrobial cystic‐fibrosis community model, hypermutable *P. aeruginosa* lineages were favoured in the absence of other bacterial species but lost this advantage in their presence. This trade‐off was associated with the fact that mutations reducing QS and QS‐associated toxin production could be beneficial under simplified abiotic conditions but disadvantageous during interspecific competition (Luján et al. 2022). Consistently, the mutation frequency of *P. aeruginosa* populations in cystic‐fibrosis samples was negatively associated with both the occurrence and diversity of co‐infecting bacteria. Together, these findings indicate that QS‐derived modification of host‐associated or environmental conditions can shape the persistence of neighbouring populations, while the resulting community context feeds back to determine the evolutionary maintenance of QS‐regulated ecological traits (Goh et al. 2023; Luján et al. 2022).

**TABLE 1:** Environmental contexts, ecological strategies and relay architectures of QS‐mediated interactions.

Microbial system	Habitat	Ecological strategy	Relay pathway	
*Streptococcus oralis* – *Actinomyces naeslundii*	Saliva‐fed oral biofilm	Shared signal accessibility	AI‐2 produced by *S. oralis* supports concentration‐dependent mutualistic biofilm growth with *A. naeslundii*	(Rickard et al. 2006)
*Enterococcus faecalis* – *Escherichia coli*	Mixed biofilm	Shared signal accessibility	*E. faecalis* ‐derived AI‐2 maintains Lsr‐dependent responsiveness in *E. coli* , promoting chemotaxis and mixed‐biofilm initiation.	(Laganenka and Sourjik 2018)
*Blautia obeum*– *Vibrio cholerae*	Recovery‐phase gut community	Asymmetric cue responsiveness	Community‐derived AI‐2 represses *V. cholerae* colonization and virulence factors, restricting pathogen establishment.	(Hsiao et al. 2014)
*Pseudomonas aeruginosa* – *Burkholderia multivorans*	Polymicrobial infection and defined casein coculture	Reciprocal coordination and resource monopolization	Cross‐recognition of AHLs couples QS states; Las‐controlled elastase opens casein, whereas HCN, rhamnolipids and phenazines restrict access to released nutrients.	(Slinger et al. 2024; Smalley et al. 2015)
*Acinetobacter baumannii* – *P. aeruginosa*	Dual‐species biofilm model	Reciprocal coordination	Heterologous AHL responsiveness restores or enhances biofilm formation in vitro.	(Bhargava et al. 2012)
AHL‐producing rumen microorganisms–EHEC	Bovine rumen	Asymmetric cue responsiveness	SdiA senses exogenous AHLs and adjusts virulence and acid‐resistance programmes associated with rumen colonization.	(Hughes et al. 2010)
AHL‐producing soft‐rot bacteria– *Pectobacterium wasabiae*	Plant lesion	Asymmetric cue responsiveness	Heterologous AHLs activate ExpR2 and rsmB, enabling earlier induction of plant‐cell‐wall‐degrading functions.	(Valente et al. 2017)
3OC6‐HSL‐producing leaf epiphytes– *Pseudomonas syringae*	Leaf phyllosphere	Asymmetric cue responsiveness	Epiphyte‐derived 3OC6‐HSL prematurely activates *P. syringae* QS, suppressing motility and leaf lesion formation.	(Dulla and Lindow 2009)
*Vibrio cholerae* –enteropathogenic *Escherichia coli*	Intestinal colonization model	Asymmetric cue responsiveness	*V. cholerae* ‐derived CAI‐1 elicits a virulence‐associated response in EPEC	(Gorelik et al. 2019)
*Stenotrophomonas maltophilia* – *P. aeruginosa*	Mixed biofilm	Asymmetric cue responsiveness	DSF is detected by PA1396, altering biofilm architecture and increasing polymyxin tolerance.	(Ryan et al. 2008)
*Streptococcus pneumoniae* lineages	Nasopharyngeal community	Asymmetric cue responsiveness	Polymorphic BlpC peptides and BlpH receptors permit non‐cognate sensing and earlier bacteriocin induction.	(Miller et al. 2018)
*Phaeobacter gallaeciensis* –susceptible marine neighbours	Marine surface‐associated patches	Conditional colonization	AHL‐QS activates TDA biosynthesis; TDA suppresses susceptible neighbours and reinforces a sessile producer state.	(Berger et al. 2011; Beyersmann et al. 2017)
*Burkholderia thailandensis* – *Chromobacterium subtsugae*	Soil‐associated coculture	Competitive feedback	BtaR2–BtaI2 activates bactobolin, while the receiver QS system increases efflux‐pump‐mediated resistance.	(Loo et al. 2023; Duerkop et al. 2009; Evans et al. 2018)
*P. aeruginosa* – *Staphylococcus aureus*	Chronic airway infection	Metabolic selection	PQS‐network‐associated HQNO inhibits respiration and selects persistent small‐colony variants in *S. aureus* .	(Hoffman et al. 2006; Rampioni et al. 2016; Fugère et al. 2014)
Competent–non‐competent *S. pneumoniae* populations	Nasopharyngeal biofilm	Genetic resource acquisition	CSP‐driven competence induces CbpD‐mediated fratricide, releasing homologous DNA for transformation.	(Steinmoen et al. 2003; Kausmally et al. 2005; Wei and Håvarstein 2012)
*V. cholerae* –non‐immune chitin‐community members	Chitinous aquatic surfaces	Genetic resource acquisition	QS and chitin sensing activate natural competence and T6SS killing, coupling DNA release to uptake.	(Borgeaud et al. 2015; Jaskólska et al. 2018; Thomas et al. 2017)
*Serratia liquefaciens* –tomato– *Alternaria alternata*	Rhizosphere to phyllosphere	Host‐mediated effects	Root AHL production primes tomato defence, reducing a spatially separated foliar fungal pathogen.	(Schuhegger et al. 2006)
*Sinorhizobium meliloti* – *Arabidopsis thaliana* – *P. syringae*	Plant‐associated root‐to‐leaf system	Host‐mediated effects	Oxo‐C14‐HSL‐producing rhizobia induce host resistance that constrains a foliar bacterial pathogen.	(Zarkani et al. 2013)
*Serratia plymuthica* HRO‐C48–plant–fungal pathogens	Rhizosphere and challenged aerial tissues	Host‐mediated effects	AHL signalling supports systemic resistance against *Botrytis cinerea* and protection from *Pythium aphanidermatum* .	(Pang et al. 2009)
Soft‐rot *Pectobacterium*– *Salmonella enterica* / EHEC	Tomato and leafy‐tissue lesions	Host‐associated niche restructuring	AHL‐QS‐dependent tissue maceration creates a nutrient‐rich lesion niche that supports enteric bacterial proliferation.	(Liu et al. 2008; George et al. 2018; Kwan et al. 2013)
*Dickeya dadantii* – *Escherichia coli* O157:H7	Postharvest lettuce leaves	Host‐associated niche restructuring	Vfm QS‐regulated pectate lyases macerate lettuce tissue; the altered lesion niche promotes *E. coli* O157:H7 proliferation.	(Banerjee et al. 2022; Yamazaki et al. 2011)
*P. aeruginosa* – *Klebsiella pneumoniae* / *Pseudomonas protegens*– *Tetrahymena pyriformis*	Mixed‐species biofilm	Associational resistance	Rhamnolipids and PQS suppress protozoan grazing, allowing otherwise grazing‐sensitive neighbours to persist.	(Goh et al. 2023)

*Note:* Table 1 summarizes the mechanistically resolved examples discussed in this Review. Each case is placed within its relevant habitat and classified according to the ecological strategy supported by quorum sensing and the relay architecture through which QS activity in the source population affects another population. The classification reflects the dominant relay architecture described for each system, while recognizing that individual interactions may combine direct signal perception, QS‐regulated chemical effectors, and host‐ or environment‐mediated consequences. Additional examples that meet the same inclusion criteria but are not discussed in detail in the main text are compiled in Table S1.

## 5 Future Perspectives and Recommendations

The propagation framework developed here provides a basis for moving from catalogues of QS‐regulated phenotypes towards questions about how ecological effects are realized in multispecies communities. Table 1 provides a comparative synthesis of the systems discussed in this Review, linking each microbial interaction to its habitat, ecological strategy, and dominant relay architecture. A central limitation of the current evidence base is that many QS‐mediated interactions have been resolved in pairwise cultures or simplified model systems, whereas natural communities contain spatially segregated populations, fluctuating resources, and multiple potential receivers. Under such conditions, the same molecular interaction may be strengthened, buffered, or redirected by local community architecture. Experiments with synthetic and complex communities have shown that spatial partitioning can alter both the type and strength of interpopulation interactions and, consequently, community diversity (Wu et al. 2022). Future work should therefore ask not only whether a QS system is present but also which source population, receiver population, and ecological intermediate are connected within a defined habitat.

This question is inseparable from environmental heterogeneity. In soil and rhizosphere habitats, pore connectivity, discontinuous water films, microscale oxygen gradients, and pulsed root‐derived substrates generate chemically distinct microsites; these conditions can retain metabolites locally while making population contact and resource accessibility highly patchy (Philippot et al. 2024; Darch et al. 2018). In marine systems, bulk‐water dilution can rapidly disperse diffusible compounds, but particles, phycospheres, and biofilms create transient nutrient‐rich patches in which cells and their metabolites remain concentrated (Raina et al. 2022). Host‐associated habitats add mucus structure, immune‐derived oxidants, nutrient release, and antimicrobial gradients, thereby modifying both the persistence of signals and the ecological consequences of QS‐regulated traits. These contrasts lead to testable predictions: direct signal responsiveness should be favoured when compatible populations repeatedly co‐localize; chemical mediation should be favored when secreted effectors persist at biologically active concentrations; and host‐ or environment‐mediated propagation should become prominent when a stable environmental intermediate can retain, amplify, or redirect the effect of a QS‐regulated trait. The observation that some QS systems operate over only a few micrometres further emphasizes that the relevant spatial scale is determined by signal uptake, receiver distribution, and the phenotype being regulated, rather than by habitat size alone (Van Gestel et al. 2021).

The three propagation modes should also be treated as potentially combinable ecological processes rather than fixed categories assigned permanently to a microbial pair. In a laboratory coculture model of the soil‐associated bacteria *Burkholderia thailandensis* and *Chromobacterium violaceum* , heterologous AHL perception by a promiscuous receptor provides an example of direct heterologous signal perception that can alter competitive timing (Chandler et al. 2012). A related study with *Chromobacterium subtsugae* further showed that non‐cognate AHLs produced by *B. thailandensis* activate the CviR receptor and induce *hcnABC* expression, converting heterologous signal responsiveness into hydrogen‐cyanide‐mediated competition (Loo et al. 2023). This sequence illustrates a broader possibility: an interaction can begin with receiver‐level cue responsiveness and subsequently acquire a chemically mediated ecological consequence. Time‐resolved studies are now needed to establish when direct sensing remains a cue, when it is translated into an extracellular effector, and when that effector subsequently changes the host or local environment experienced by additional populations.

Addressing these questions requires methods that resolve identity, location, activity, and chemical exposure in the same community. Spatial metatranscriptomics can simultaneously map bacterial, fungal, and host transcriptional states in tissue sections, as demonstrated for outdoor‐grown *Arabidopsis thaliana* leaves at 55‐μm resolution (Saarenpää et al. 2024). This approach does not by itself establish QS activity, but when combined with genetically encoded QS reporters, targeted signal quantification, and imaging mass spectrometry, it can identify where candidate source populations, receivers, and host responses co‐occur. Stable‐isotope approaches add a complementary functional dimension. Single‐cell stable‐isotope probing can track substrate incorporation at cellular resolution (Alcolombri et al. 2022), whereas flow‐through stable‐isotope probing reduces cross‐feeding and helps distinguish primary consumers from populations responding to released metabolic products (Mooshammer et al. 2021). Genome‐resolved quantitative stable‐isotope probing can further connect environmental conditions with the growth and functional potential of active taxa in natural soils (Greenlon et al. 2022). Together, these approaches provide a route from inferred network edges to spatially and metabolically resolved interaction hypotheses.

Future experiments should integrate these tools into matched perturbation designs. A useful minimum design would compare a QS‐defective source strain, an effector‐defective source strain, and an appropriately complemented control; quantify receiver transcriptional or physiological responses; and simultaneously measure the relevant signal, mediator, and habitat variable over time. Where feasible, receiver mutants or reporter strains should be included to determine whether a response depends on signal detection, susceptibility to an effector, or a change in the shared environment. Such designs would distinguish direct signal responses from downstream chemical or environmental relays and reveal whether the same route operates across spatial scales. The micron‐scale analysis of QS‐regulated horizontal gene transfer provides a useful precedent: it linked a defined signalling architecture, local receiver composition, and a measurable ecological output within structured communities (Van Gestel et al. 2021).

Finally, the framework generates a set of ecological and evolutionary predictions. If environmental conditions determine the costs and benefits of QS‐regulated traits, then they should also determine whether signalling, signal responsiveness, and downstream effectors are retained, diversified, or lost. Recent experimental‐evolution work with *Pseudomonas aeruginosa* showed that oxidative stress together with abundant free amino acids in inflammation‐like environments constrained the loss of QS, demonstrating that nutritional conditions alone do not predict selection on QS regulation (Fu et al. 2025). Comparative experiments across hydration, flow, nutrient, and host‐stress gradients could therefore test whether particular environmental architectures repeatedly favour coordination, asymmetric cue responsiveness, chemical competition, or host‐ and environment‐mediated effects. In applied settings, this framework may guide microbiome engineering by identifying the propagation step and environmental condition most closely linked to a desired community outcome, rather than treating every QS system as an isolated molecular target.

## 6 Conclusions

Quorum sensing has traditionally been interpreted as a cell‐density‐dependent communication mechanism through which microorganisms coordinate collective behaviours using diffusible signalling molecules. While this perspective has greatly advanced our understanding of QS chemistry, regulatory networks, and physiological functions, the ecological consequences of QS remain fragmented across studies focusing on individual signals, microbial taxa, or specific interaction outcomes. Viewing QS solely as communication between neighbouring cells therefore provides an incomplete picture of how QS activity in one population can influence other populations within complex microbial communities. A QS event can affect another microbial population through direct perception of the source signal itself, through exposure to a QS‐regulated chemical effector, or through a host or environmental condition modified by QS activity. These relay architectures are distinguished by the component that connects QS activity in one population to its effect on another population, including the source signal, a QS‐regulated chemical effector, or a modified shared host or environmental condition.

In this Review, we use these relay architectures as the first dimension of a framework for organizing the effects of QS activity on microbial interactions. The second dimension identifies the ecological strategies associated with each architecture.

Across these relay architectures, microbial populations can exhibit distinct ecological strategies. Direct perception of shared or heterologous signals can be associated with shared signal accessibility, reciprocal coordination, or asymmetric cue responsiveness where populations are co‐localized. QS‐regulated effectors can support interference, resource capture, and metabolic selection when neighbouring populations share chemically connected microsites. Host‐ and environment‐mediated routes can extend these consequences beyond direct molecular contact, altering the establishment, persistence, or competitive performance of other populations through altered host states, predator pressures, or habitat conditions. Together, these propagation routes illustrate that the ecological influence of QS extends beyond direct signal perception and operates across multiple levels of biological organization, from individual populations to entire microbial ecosystems.

As research continues to address increasingly complex natural microbial ecosystems, determining how QS activity in one population becomes linked to effects on others within heterogeneous communities will be essential for connecting molecular regulation with ecological function. The framework therefore generates experimentally testable predictions about when particular QS‐associated interactions should emerge. Spatial confinement, signal uptake, matrix structure, nutrient heterogeneity, host physiology, and temporal variation are expected to influence whether a QS response remains local, is translated into a chemical ecological effect, or is amplified through environmental feedback. Resolving these predictions will require experiments that measure source activity, mediator dynamics, receiver responses, and ecological outcomes in the same spatially structured system. Ultimately, the framework directs attention to the source activity, intervening components, and receiver‐ or community‐level outcomes that together determine the ecological consequences of QS, facilitating comparisons across ecosystems, promoting integration between molecular microbiology and microbial ecology, and providing conceptual guidance for predicting and manipulating microbiome functions.

## Author Contributions

**Changchang Liang:** writing – original draft, visualization, investigation, writing – review and editing, conceptualization. **Danlei Chen:** investigation, conceptualization, writing – review and editing. **Shuyang Sun:** writing – review and editing, visualization. **Aidong Yang:** investigation, supervision, methodology, writing – review and editing, project administration. **Jianjun Qiao:** project administration, resources, writing – review and editing, supervision. **Shengbo Wu:** conceptualization, writing – review and editing, funding acquisition, project administration, supervision, visualization.

## Funding

This work was supported by the National Key Research and Development Program of China (2024YFA0920200) and National Natural Science Foundation of China (32300022).

## Conflicts of Interest

The authors declare no conflicts of interest.

## Supporting information


**Table S1:** Environmental contexts, ecological strategies and relay architectures of QS‐mediated interactions.

## Data Availability

Data sharing not applicable to this article as no datasets were generated or analysed during the current study.
