# Supplementary material for: Relay Architectures and Ecological Strategies of Quorum Sensing Effects on Microbial Interactions
Source: Environ Microbiol. 2026 Oct 11;28(10):e70440. doi: 10.1111/1462-2920.70440 (PMC13654649; doi:10.1111/1462-2920.70440)
Supplement: Supplementary file 1 [file EMI-28-e70440-s001.pdf]

Table S1 | Environmental contexts, ecological strategies and relay architectures of QS-mediated interactions

The table consolidates named systems discussed in the revised manuscript and additional external cases meeting the same source-relay-receiver criteria. Examples are organized by their principal relay architecture; some interactions combine more than one architecture.

Microbial system	Habitat	Relay architecture	Ecological strategy	Relay pathway	Key reference(s)
**Examples discussed in the manuscript**					
Streptococcus oralis–*Actinomyces naeslundii*	Saliva-fed oral biofilm	Direct signal perception	Shared signal accessibility	AI-2 produced by *S. oralis* supports concentration-dependent mutualistic biofilm growth with *A. naeslundii*.	Rickard et al. (2006)
Enterococcus faecalis–*Escherichia coli*	Mixed biofilm	Direct signal perception	Shared signal accessibility	*E. faecalis*-derived AI-2 maintains Lsr-dependent responsiveness in *E. coli*, promoting chemotaxis and mixed-biofilm initiation.	Laganenka and Sourjik (2018)
Blautia obeum–*Vibrio cholerae*	Recovery-phase gut community	Direct signal perception	Asymmetric cue responsiveness	Community-derived AI-2 represses *V. cholerae* colonization and virulence factors, restricting pathogen establishment.	Hsiao et al. (2014)
Pseudomonas aeruginosa–*Burkholderia multivorans*	Polymicrobial infection and defined casein coculture	Direct signal perception + QS-regulated chemical effector	Asymmetric cue responsiveness	Cross-recognition of AHLs couples QS states; Las-controlled elastase opens casein, whereas HCN, rhamnolipids and phenazines restrict access to released nutrients.	Slinger et al. (2024); Smalley et al. (2015)
Acinetobacter baumannii–*P. aeruginosa*	Dual-species biofilm model	Direct signal perception	Reciprocal coordination	Heterologous AHL responsiveness restores or enhances biofilm formation in vitro.	Bhargava et al. (2012)
AHL-producing rumen microorganisms–EHEC	Bovine rumen	Direct signal perception	Asymmetric cue responsiveness	SdiA senses exogenous AHLs and adjusts virulence and acid-resistance programmes associated with rumen colonization.	Hughes et al. (2010)
AHL-producing soft-rot bacteria–*Pectobacterium wasabiae*	Plant lesion	Direct signal perception	Asymmetric cue responsiveness	Heterologous AHLs activate ExpR2 and rsmB, enabling earlier induction of plant-cell-wall-degrading functions.	Valente et al. (2017)
3OC6-HSL-producing leaf epiphytes–*Pseudomonas syringae*	Leaf phyllosphere	Direct signal perception	Asymmetric cue responsiveness	Epiphyte-derived 3OC6-HSL prematurely activates *P. syringae* QS, suppressing motility and leaf lesion formation.	Dulla and Lindow (2009)
Vibrio cholerae–enteropathogenic *Escherichia coli*	Intestinal colonization model	Direct signal perception	Asymmetric cue responsiveness	*V. cholerae*-derived CAI-1 is sensed by EPEC and enhances its virulence programme.	Gorelik et al. (2019)
Stenotrophomonas maltophilia–*P. aeruginosa*	Mixed biofilm	Direct signal perception	Asymmetric cue responsiveness	DSF is detected by PA1396, altering biofilm architecture and increasing polymyxin tolerance.	Ryan et al. (2008)
Streptococcus pneumoniae lineages	Nasopharyngeal community	Direct signal perception	Asymmetric cue responsiveness	Polymorphic BlpC peptides and BlpH receptors permit non-cognate sensing and earlier bacteriocin induction.	Miller et al. (2018)
Phaeobacter gallaeciensis–susceptible marine neighbours	Marine surface-associated patches	Mediation by QS-regulated effectors	Conditional colonization	AHL-QS activates TDA biosynthesis; TDA suppresses susceptible neighbours and reinforces a sessile producer state.	Berger et al. (2011); Beyersmann et al. (2017)
Burkholderia thailandensis–*Chromobacterium subtsugae*	Soil-associated coculture	Mediation by QS-regulated effectors	Competitive feedback	BtaR2–BtaI2 activates bactobolin, while the receiver QS system increases efflux-pump-mediated resistance.	Loo et al. (2023); Duerkop et al. (2009); Evans et al. (2018)
P. aeruginosa–*Staphylococcus aureus*	Chronic airway infection	Mediation by QS-regulated effectors	Metabolic selection	PQS-network-associated HQNO inhibits respiration and selects persistent small-colony variants in *S. aureus*.	Hoffman et al. (2006); Rampioni et al. (2016); Fugère et al. (2014)
Competent–non-competent *S. pneumoniae* populations	Nasopharyngeal biofilm	Mediation by QS-regulated effectors	Genetic resource acquisition	CSP-driven competence induces CbpD-mediated fratricide, releasing homologous DNA for transformation.	Steinmoen et al. (2003); Kausmally et al. (2005); Wei and Håvarstein (2012)

Microbial system	Habitat	Relay architecture	Ecological strategy	Relay pathway	Key reference(s)
V. cholerae–non-immune chitin-community members	Chitinous aquatic surfaces	Mediation by QS-regulated effectors	Genetic resource acquisition	QS and chitin sensing activate natural competence and T6SS killing, coupling DNA release to uptake.	Borgeaud et al. (2015); Jaskólska et al. (2018); Thomas et al. (2017)
Serratia liquefaciens–tomato–*Alternaria alternata*	Rhizosphere to phyllosphere	Relays through QS-modified host and environmental conditions	Host-mediated effects	Root AHL production primes tomato defence, reducing a spatially separated foliar fungal pathogen.	Schuhegger et al. (2006)
Sinorhizobium meliloti–*Arabidopsis thaliana*–*P. syringae*	Plant-associated root-to-leaf system	Relays through QS-modified host and environmental conditions	Host-mediated effects	oxo-C14-HSL-producing rhizobia induce host resistance that constrains a foliar bacterial pathogen.	Zarkani et al. (2013)
Serratia plymuthica HRO-C48–plant–fungal pathogens	Rhizosphere and challenged aerial tissues	Relays through QS-modified host and environmental conditions	Host-mediated effects	AHL signalling supports systemic resistance against *Botrytis cinerea* and protection from *Pythium aphanidermatum*.	Pang et al. (2009)
Soft-rot *Pectobacterium*–*Salmonella enterica* / EHEC	Tomato and leafy-tissue lesions	Relays through QS-modified host and environmental conditions	Host-associated niche restructuring	AHL-QS-dependent tissue maceration creates a nutrient-rich lesion niche that supports enteric bacterial proliferation.	Liu et al. (2008); George et al. (2018); Kwan et al. (2013)
Dickeya dadantii–*Escherichia coli* O157:H7	Postharvest lettuce leaves	Relays through QS-modified host and environmental conditions	Host-associated niche restructuring	Vfm QS-regulated pectate lyases macerate lettuce tissue; the altered lesion niche promotes *E. coli* O157:H7 proliferation.	Banerjee et al. (2022); Yamazaki et al. (2011)
P. aeruginosa–*Klebsiella pneumoniae* / *Pseudomonas protegens*–*Tetrahymena pyriformis*	Mixed-species biofilm	Relays through QS-modified host and environmental conditions	Associational resistance	Rhamnolipids and PQS suppress protozoan grazing, allowing otherwise grazing-sensitive neighbours to persist.	Goh et al. (2023)
**Additional examples not discussed in detail in the manuscript**					
Escherichia coli–*Vibrio cholerae*	Zebrafish hindbrain ventricle infection model	Direct signal perception	Asymmetric cue responsiveness	*E. coli*-derived AI-2 induces *V. cholerae* aggregate disassembly, increasing interspecies contact and T6SS-mediated killing in vivo.	Virgo et al. (2026)
Pantoea agglomerans 9Rz4–phytopathogenic fungi	Rhizosphere-derived isolate	Mediation by QS-regulated effectors	Chemical antagonism	The AHL-QS system modulates herbicolin A gene-cluster expression; herbicolin A mediates broad antifungal activity.	Matilla et al. (2023)
Ralstonia solanacearum–*Fusarium oxysporum*	Soil-borne plant-associated system	Mediation by QS-regulated effectors	Interkingdom phenotypic modulation	PhcBSR quorum sensing regulates ralsolamycin biosynthesis, which alters *F. oxysporum* mycelial growth and chlamydospore formation	Li et al. (2017, 2022)
Aeromonas sp. GLY-2107–*Microcystis aeruginosa*	Lake Taihu freshwater bloom	Mediation by QS-regulated effectors	Chemical antagonism	C4-HSL-mediated QS positively regulates 3-methylindole and negatively regulates cyclo(Gly-Phe) production, thereby modulating algicidal activity	Guo et al. (2016)
Bacillus sp. S51107–*Microcystis aeruginosa*	Lake Taihu freshwater bloom	Mediation by QS-regulated effectors	Chemical antagonism	NprR–NprX peptide QS regulates production of high-molecular-weight extracellular algicidal substance(s) active against *M. aeruginosa*	Wu et al. (2017)

Reference list

[16] Rickard AH, Palmer RJ, Blehert DS et al. Autoinducer 2: a concentration-dependent signal for mutualistic bacterial biofilm growth. *Molecular Microbiology* 2006;60(6):1446-56. https://doi.org/10.1111/j.1365-2958.2006.05202.x.

[17] Laganenka L, Sourjik V. Autoinducer 2-Dependent Escherichia coli Biofilm Formation Is Enhanced in a Dual-Species Coculture. *Appl Environ Microbiol* 2018;84(5):e02638-17. https://doi.org/10.1128/AEM.02638-17.

[18] Hsiao A, Ahmed AMS, Subramanian S et al. Members of the human gut microbiota involved in recovery from Vibrio cholerae infection. Nature 2014;515(7527):423-6. https://doi.org/10.1038/nature13738.

[19] Slinger BL, Banerjee S, Chandler JR et al. Interspecies Crosstalk via LuxI/LuxR-Type Quorum Sensing Pathways Contributes to Decreased Nematode Survival in Coinfections of *Pseudomonas aeruginosa* and *Burkholderia multivorans*. *ACS Chemical Biology* 2024;19(12):2557-68. https://doi.org/10.1021/acschembio.4c00641.

[20] Bhargava N, Sharma P, Capalash N. N-acyl homoserine lactone mediated interspecies interactions between *A. baumannii* and *P. aeruginosa*. *Biofouling* 2012;28(8):813-22. https://doi.org/10.1080/08927014.2012.714372.

[21] Hughes DT, Terekhova DA, Liou L et al. Chemical sensing in mammalian host-bacterial commensal associations. *Proc Natl Acad Sci USA* 2010;107(21):9831-6. https://doi.org/10.1073/pnas.1002551107.

[22] Valente RS, Nadal-Jimenez P, Carvalho AFP et al. Signal Integration in Quorum Sensing Enables Cross-Species Induction of Virulence in *Pectobacterium wasabiae*. *mBio* 2017;8(3):e00398-17. https://doi.org/10.1128/mBio.00398-17.

[23] Dulla GFJ, Lindow SE. Acyl-homoserine lactone-mediated cross talk among epiphytic bacteria modulates behavior of *Pseudomonas syringae* on leaves. *The ISME Journal* 2009;3(7):825-34. https://doi.org/10.1038/ismej.2009.30.

[24] Gorelik O, Levy N, Shaulov L et al. *Vibrio cholerae* autoinducer-1 enhances the virulence of enteropathogenic *Escherichia coli*. *Sci Rep* 2019;9(1):4122. https://doi.org/10.1038/s41598-019-40859-1.

[25] Ryan RP, Fouhy Y, Garcia BF et al. Interspecies signalling via the *Stenotrophomonas maltophilia* diffusible signal factor influences biofilm formation and polymyxin tolerance in *Pseudomonas aeruginosa*. *Molecular Microbiology* 2008;68(1):75-86. https://doi.org/10.1111/j.1365-2958.2008.06132.x.

[26] Miller EL, Kjos M, Abrudan MI et al. Eavesdropping and crosstalk between secreted quorum sensing peptide signals that regulate bacteriocin production in *Streptococcus pneumoniae*. *The ISME Journal* 2018;12(10):2363-75. https://doi.org/10.1038/s41396-018-0178-x.

[27] Berger M, Neumann A, Schulz S et al. Tropodithietic Acid Production in *Phaeobacter gallaeciensis* Is Regulated by N-Acyl Homoserine Lactone-Mediated Quorum Sensing. *Journal of Bacteriology* 2011;193(23):6576-85. https://doi.org/10.1128/JB.05818-11.

[28] Beyersmann PG, Tomasch J, Son K et al. Dual function of tropodithietic acid as antibiotic and signaling molecule in global gene regulation of the probiotic bacterium *Phaeobacter inhibens*. *Sci Rep* 2017;7(1):730. https://doi.org/10.1038/s41598-017-00784-7.

[29] Urvoy M, Labry C, L'Helguen S et al. Quorum Sensing Regulates Bacterial Processes That Play a Major Role in Marine Biogeochemical Cycles. *Front Mar Sci* 2022;9:834337. https://doi.org/10.3389/fmars.2022.834337.

[30] Loo C, Koirala P, Smith NC et al. Cross-species activation of hydrogen cyanide production by a promiscuous quorum-sensing receptor promotes *Chromobacterium subtsugae* competition in a dual-species model. *Microbiology* 2023;169(2). https://doi.org/10.1099/mic.0.001294.

[31] Duerkop BA, Varga J, Chandler JR et al. Quorum-Sensing Control of Antibiotic Synthesis in *Burkholderia thailandensis*. *J Bacteriol* 2009;191(12):3909-18. https://doi.org/10.1128/JB.00200-09.

[32] Evans KC, Benomar S, Camuy-Velez LA et al. Quorum-sensing control of antibiotic resistance stabilizes cooperation in *Chromobacterium violaceum*. *The ISME Journal* 2018;12(5):1263-72. https://doi.org/10.1038/s41396-018-0047-7.

[33] Hoffman LR, Déziel E, D'Argenio DA et al. Selection for Staphylococcus aureus small-colony variants due to growth in the presence of *Pseudomonas aeruginosa*. *Proc Natl Acad Sci USA* 2006;103(52):19890-5. https://doi.org/10.1073/pnas.0606756104.

[34] Rampioni G, Falcone M, Heeb S et al. Unravelling the Genome-Wide Contributions of Specific 2-Alkyl-4-Quinolones and PqsE to Quorum Sensing in *Pseudomonas aeruginosa*. *PLoS Pathog* 2016;12(11):e1006029. https://doi.org/10.1371/journal.ppat.1006029.

[35] Fugere A, Lalonde Séguin D, Mitchell G et al. Interspecific Small Molecule Interactions between Clinical Isolates of *Pseudomonas aeruginosa* and *Staphylococcus aureus* from Adult Cystic Fibrosis Patients. *PLoS ONE* 2014;9(1):e86705. https://doi.org/10.1371/journal.pone.0086705.

[36] Msimbira LA, Balimponya EG, Mwakatoga JD et al. Rhizosphere Intelligence: Harnessing Root-Microbe Dynamics for Resilient Cropping System. *In: Aide M, Braden I, Nakasagga S et al.* (eds), *Agricultural Sciences*, vol. 42. n.p.: IntechOpen, 2026. https://doi.org/10.5772/intechopen.1013508.

[37] Smalley NE, An D, Parsek MR et al. Quorum Sensing Protects *Pseudomonas aeruginosa* against Cheating by Other Species in a Laboratory Coculture Model. *J Bacteriol* 2015;197(19):3154-9. https://doi.org/10.1128/JB.00482-15.

[38] Steinmoen H, Teigen A, Håvarstein LS. Competence-Induced Cells of *Streptococcus pneumoniae* Lyse Competence-Deficient Cells of the Same Strain during Cocultivation. *J Bacteriol* 2003;185(24):7176-83. https://doi.org/10.1128/JB.185.24.7176-7183.2003.

[39] Kausmally L, Johnsborg O, Lunde M et al. Choline-Binding Protein D (CbpD) in *Streptococcus pneumoniae* Is Essential for Competence-Induced Cell Lysis. *J Bacteriol* 2005;187(13):4338-45. https://doi.org/10.1128/JB.187.13.4338-4345.2005.

[40] Wei H, Håvarstein LS. Fratricide Is Essential for Efficient Gene Transfer between *Pneumococci* in Biofilms. *Appl Environ Microbiol* 2012;78(16):5897-905. https://doi.org/10.1128/AEM.01343-12.

[41] Borgeaud S, Metzger LC, Scrignari T et al. The type VI secretion system of *Vibrio cholerae* fosters horizontal gene transfer. *Science* 2015;347(6217):63-67. https://doi.org/10.1126/science.1260064.

[42] Jaskolska M, Stutzmann S, Stoudmann C et al. QstR-dependent regulation of natural competence and type VI secretion in *Vibrio cholerae*. *Nucleic Acids Research* published online 8 Aug. 2018. https://doi.org/10.1093/nar/gky717.

[43] Thomas J, Watve SS, Ratcliff WC et al. Horizontal Gene Transfer of Functional Type VI Killing Genes by Natural Transformation. *mBio* 2017;8(4):e00654-17. https://doi.org/10.1128/mBio.00654-17.

[44] Schuhegger R, Ihring A, Gantner S et al. Induction of systemic resistance in tomato by N-acyl-L-homoserine lactone-producing rhizosphere bacteria. *Plant Cell & Environment* 2006;29(5):909-18. https://doi.org/10.1111/j.1365-3040.2005.01471.x.

[45] Zarkani A, Stein E, Rohrich C et al. Homoserine Lactones Influence the Reaction of Plants to *Rhizobia*. *IJMS* 2013;14(8):17122-46. https://doi.org/10.3390/ijms140817122.

[46] Pang Y, Liu X, Ma Y et al. Induction of systemic resistance, root colonisation and biocontrol activities of the rhizospheric strain of *Serratia plymuthica* are dependent on N-acyl homoserine lactones. *European Journal of Plant Pathology* 2009;124(2):261-8. https://doi.org/10.1007/s10658-008-9411-1.

[47] Liu H, Coulthurst SJ, Pritchard L et al. Quorum Sensing Coordinates Brute Force and Stealth Modes of Infection in the Plant Pathogen *Pectobacterium atrosepticum*. *PLoS Pathog* 2008;4(6):e1000093. https://doi.org/10.1371/journal.ppat.1000093.

[48] George AS, Cox CE, Desai P et al. Interactions of Salmonella enterica *Serovar Typhimurium* and *Pectobacterium carotovorum* within a Tomato Soft Rot. *Appl Environ Microbiol* 2018;84(5):e01913-17. https://doi.org/10.1128/AEM.01913-17.

[49] Kwan G, Charkowski AO, Barak JD. *Salmonella enterica* Suppresses *Pectobacterium carotovorum* subsp. carotovorum Population and Soft Rot Progression by Acidifying the Microaerophilic Environment. *mBio* 2013;4(1):e00557-12. https://doi.org/10.1128/mBio.00557-12.

[50] Banerjee B, Zeng Q, Yu M et al. Quorum-Sensing Master Regulator VfmE Is a c-di-GMP Effector That Controls Pectate Lyase Production in the Phytopathogen Dickeya dadantii. *Microbiol Spectr* 2022;10(2):e01805-21. https://doi.org/10.1128/spectrum.01805-21.

[51] Yamazaki A, Li J, Hutchins WC et al. Commensal Effect of Pectate Lyases Secreted from *Dickeya dadantii* on Proliferation of *Escherichia coli* O157:H7 EDL933 on Lettuce Leaves. *Appl Environ Microbiol* 2011;77(1):156-62. https://doi.org/10.1128/AEM.01079-10.

[52] Goh YF, Roder HL, Chan SH et al. Associational Resistance to Predation by Protists in a Mixed Species Biofilm. *Appl Environ Microbiol* 2023;89(2):e01741-22. https://doi.org/10.1128/aem.01741-22.

[53] Lujan AM, Paterson S, Hesse E et al. Polymicrobial infections can select against *Pseudomonas aeruginosa* mutators because of quorum-sensing trade-offs. *Nature Ecology & Evolution* 2022;6(7):979-88. https://doi.org/10.1038/s41559-022-01768-1.

[54] Wu F, Ha Y, Weiss A et al. Modulation of microbial community dynamics by spatial partitioning. *Nature Chemical Biology* 2022;18(4):394-402. https://doi.org/10.1038/s41589-021-00961-w.

Additional references used in Table S1

Virgo M, Painter H, Xue S et al. Interspecies quorum sensing signals modulate multicellular organization and enhance contact-dependent antagonism in *Vibrio cholerae*. *Nature Communications* 2026. https://doi.org/10.1038/s41467-026-74086-w.

Matilla MA, Evans TJ, Martín J et al. Herbicolin A production and its modulation by quorum sensing in a *Pantoea agglomerans* rhizobacterium bioactive against a broad spectrum of plant-pathogenic fungi. *Microbial Biotechnology* 2023;16(8):1690–1700. https://doi.org/10.1111/1751-7915.14193.

Li P, Yin W, Yan J et al. Modulation of inter-kingdom communication by PhcBSR quorum sensing system in *Ralstonia solanacearum* phylotype I strain GMI1000. *Frontiers in Microbiology* 2017;8:1172. https://doi.org/10.3389/fmicb.2017.01172.

Li P, Cao X, Zhang L, Lv M, Zhang L-H. PhcA and PhcR regulate ralsolamycin biosynthesis oppositely in Ralstonia solanacearum. Frontiers in Plant Science 2022;13:903310. https://doi.org/10.3389/fpls.2022.903310.

Guo X, Liu X, Wu L, Pan J, Yang H. The algicidal activity of *Aeromonas* sp. strain GLY-2107 against bloom-forming Microcystis aeruginosa is regulated by N-acyl homoserine lactone-mediated quorum sensing. *Environmental Microbiology* 2016;18(11):3867–3883. https://doi.org/10.1111/1462-2920.13346.

Wu L, Guo X, Liu X, Yang H. NprR-NprX quorum-sensing system regulates the algicidal activity of *Bacillus* sp. strain S51107 against bloom-forming cyanobacterium *Microcystis aeruginosa*. *Frontiers in Microbiology* 2017;8:1968. https://doi.org/10.3389/fmicb.2017.01968.
